# Experimental Whisky Fermentations: Influence of Wort Pretreatments

**DOI:** 10.3390/foods10112755

**Published:** 2021-11-10

**Authors:** Martina Daute, Frances Jack, Barry Harrison, Graeme Walker

**Affiliations:** 1 Division of Engineering and Food Sciences, Abertay University, Dundee DD1 1HG, UK; g.walker@abertay.ac.uk; 2 The Scotch Whisky Research Institute, Edinburgh EH14 4AH, UK; fj@swri.co.uk (F.J.); bh@swri.co.uk (B.H.)

**Keywords:** wort, fermentation, flavour, spirit, whisky, congener

## Abstract

In addition to ethanol yield, the production of flavour congeners during fermentation is a major consideration for Scotch whisky producers. Experimental whisky fermentations can provide useful information to the industry, and this is the focus of this paper. This study investigated the impact of wort pretreatments (boiled, autoclaved, filtered) on fermentation performance and flavour development in Scotch whisky distillates as an alternative to freezing wort for storage. Our study showed that no significant sensorial differences were detected in low wines (first distillates), while the chemical compositions showed clear changes in increased levels of esters and higher alcohols in boiled and autoclaved wort. In contrast, filtered wort comprised overall lower levels of congeners. Regarding alcohol yield, all three pretreatments resulted in decreased yields. In practice, the pretreatment of wort prior to fermentation requires additional process operations, while freezing requires large storage units. The pretreatments adopted in this study significantly influence the composition of the malt wort used for experimental whisky fermentations, and this results in a poorer fermentation performance compared with untreated wort. We recommend the use of fresh or frozen wort as the best options for small-scale fermentation trials.

## 1. Introduction

The production of Scotch whisky is a nonsterile process. The wort is not boiled prior to the fermentation, and no antimicrobials may be used to reduce microbial contamination, nor other components used to stabilise the product [1]. The inherent microflora of the distillery, the malt, and the (frequently wooden) washbacks (fermentation vessels) are important for flavour development in Scotch whisky. In particular, lactic acid bacteria are involved in converting higher alcohols to acetate esters after alcoholic fermentation by the yeast, *Saccharomyces cerevisiae* [2,3,4,5,6,7,8]. Additionally, by not heat-treating the wort, the enzymes that breakdown the starch (α-amylase, β-amylase, limit dextrinase, α-glucosidase) stay active during the fermentation [9,10,11,12,13,14].

In recent years, interest in Scotch whisky fermentation has shifted from being mostly focused on alcohol yield, to including how the process changes influence the spirit flavour [15,16,17,18,19]. With the main aim of the distillery being to sell high-quality spirit, small-scale studies are required in order to assess changes in the process. The use of unusual (occasionally non-*Saccharomyces*) yeasts is an especially new and interesting topic, resulting in the availability of the Scotch whiskies produced with them, for example, Glen Elgin 1998 18 Year Old Special Release 2017 (using *Schizosaccharomyces pombe*) [20], and Glenmorangie Allta (using local wild yeast from the Cadboll barley, *Saccharomyces diaemath*) [21].

To improve the research of Scotch whisky fermentations, reproducible small-scale research trials must be established. A limited number of small-scale systems for whisky fermentation have been developed [22], with many techniques adapted from brewing [23]. The main issue for reliable small-scale fermentation is to get the same quality of wort for several consecutive trial fermentations. Varieties in batch or day variations, such as wort gravity, free amino nitrogen (FAN), or indigenous microorganisms, can significantly influence the fermentation and overshadow other factors researched, such as the fermentation conditions or yeast strains. To date, researchers have used three different approaches to acquire malt: producing wort in the lab with a nanobrewery; following standard brewing protocols, such as EBC methods [4,11,12,19,22,24,25,26,27,28]; or obtaining a large batch of wort from a local distillery and freezing it [29,30,31]. Nevertheless, for all three approaches, enough wort from the same batch must be obtained before starting a study, and this makes extending experiments nearly impossible. Small changes in wort composition can have an impact on the fermentation performance and congener production [32]. Another issue is that the microorganisms in the wort change from batch to batch, impacted by cleaning, raw materials, and environmental factors [8,33], which especially influences congener production and can act as a nuisance factor that is difficult to control. They can add congeners, as well as lower the pH, which can influence the experiment [34]. As a result, the question arose as to whether physical or thermal treatment can help to standardise wort. Previous research has shown that treatments impacting the microbial nutritional composition can have a significant effect on the typical Scotch whisky flavour profiles, especially on the reduction of esters [18,35]. However, the effect of different pretreatments on flavour and congener development has not been researched yet.

To assess whether, and to what magnitude, the pretreatment of wort impacts the fermentation performance and congener production, we explored the impact of the treatment methods for wort on these factors. This paper describes the fermentation effects of wort filtering, boiling, and autoclaving as alternative wort treatment methods, and how they impact ethanol yield, yeast viability, congener production, and spirit flavour.

## 2. Materials and Methods

### 2.1. Wort Collection

The wort was collected from a local Scotch malt whisky distillery. Wort gravity was determined using an Anton Paar Density Meter DMA 35 (Anton Paar, Graz, Austria); original gravity was 1070.0°, and the pH was 5.6. The wort was stored in a frozen state at −18 °C in 1 L aliquots and defrosted prior to usage.

### 2.2. Wort Pretreatment

Three pretreatments of wort were studied, namely, filtered wort, boiled wort, and autoclaved wort. The filtration of wort (FW) was carried out using a Munktell Fluted No. 11 320 mm diameter and 11/N grade filter paper (Falun, Sweden), with a particle retention of 25 µm. The boiling of wort (BW) was for 1 h on a hot plate (HT1 Halogen Hotplate, Bibby Scientific, Stone, UK), covered with a watch glass. The boiled wort was cooled down in cold tap water to room temperature, and the gravity was, with water, adjusted to 1070°. The autoclaved wort (AW) was autoclaved for 15 min at 121°C (AMB24ON Astell Scientific, Sidcup, UK). The control wort (CW) was only frozen and defrosted to room temperature before use.

### 2.3. Fermentation Setup

Small-scale fermentation was carried out in 500 mL Duran bottles containing 300 mL of pretreated wort. The fermentation was started by pitching 1 g/L DistillaMax^®^ MW (*Saccharomyces cerevisiae*) (Lallemand Biofuels & Distilled Spirits, Montreal, Canada) into the wort. The dried yeast was rehydrated as recommended by the supplier. An amount of 1 g of yeast was incubated in 10 mL of water for 5 min at room temperature before inoculation. All fermentations were performed in triplicate at the same time. At the end of the fermentation, the gravity (Anton Paar Density Meter DMA35, Anton Paar, Graz, Austria), the pH (pH meter HI 208, Hanna Instruments, Padova, Italy), and the cell count and viability with flow cytometry were measured. An amount of 260 mL of the wash was frozen at −7 °C until distillation. The ABV (percentage of alcohol by volume) in the wash was calculated by gravity as a standard method for the Beer Duty Return [36].

### 2.4. Monitoring of the Fermentation Performance with the Ankom RG Gas Production System

The fermentation bottles were sealed using Ankom RF Gas Production Systems (Ankom) (Ankom Technology, Macedon, NY, USA). These units measure gas pressure changes and allow the fermentation performance to be monitored by continuous gas production measurements. The fermentation was carried out in a temperature-controlled water bath (TXF200, Grant Instruments Ltd., Royston, UK) at 30 °C for 65 h. The Ankom data were processed as described in Black et al. (2021) [37].

### 2.5. Measuring Particle Count, Yeast Count, and Yeast Viability

The particle count, yeast count, and yeast viability were measured with a flow cytometer, Cyflow SL (Partec, Münster, Germany), and the software, Flomax (Quantum Analysis GmbH, Muenster, Germany, Version 2.82 (16 April 2012)). Fermentation samples were filtered through a CellTrics 50 µm filter (Sysmex, Kobe, Japan), followed by an appropriate dilution in Ringer solution (Oxoid, Hampshire, England), produced according to the manufacturer’s instructions, resulting in a total count of between 500–800 counts/s. The region of yeast cells was gated in an FSC/SSC diagram and was used to calculate the total cell count by considering the previous dilution factor. To determine the viability, the Yeast ControlTM-Viability kit (Sysmex, Kobe, Japan) was used, as stated by the producers. The same dilutions used for the cell count were applied. The gated yeast region in the FSC/SSC diagram was displayed in a FL1-green/FL2-red diagram. Crosstalk compensation was used to separate the groups. Yeast with green fluorescens were alive, and cells with red fluorescens were dead.

### 2.6. Photomicroscopy

Microscope pictures were taken of the four different worts and washes. A Leica DM 2000 (Leica Microsystems Ltd., Heerbrugg, Germany) microscope, with a Leica DFC225 camera Version 7.3.0.0 (Leica Microsystems Ltd., Heerbrugg, Germany), and the Leica Application Suite Version 3.7.0 (Build:681) (Leica Microsystems Ltd., Heerbrugg, Germany) were used to take pictures.

### 2.7. Production of Low Wines by Distillation

The spirits were produced by a single distillation in order to obtain “low wines”. The distillations were carried out using the copper lab-scale wash still manufactured for the Scotch Whisky Research Institute (SWRI). Frozen wash samples were defrosted in warm water. The still was cleaned and conditioned before usage by distilling a 50% water and 50% ethanol mixture on a maximum heat setting. An amount of 260 mL was poured into the wash still, and 100 mL of distillate was collected. An amount of 260 mL of the frozen wash was poured into the still with 10 PTFE boiling stones (Sigma-Aldrich 2243558-1EA, St. Louis, MO, USA), and 10 drops of EcoLab Component A Antifoam. The heating mantle (Fisher Scientific, Waltham, MA, USA) was switched on (set to 8), and 100 mL of low wines was collected. The distillate was cooled down during distillation with a WK 4600 circulation thermostat (Lauda, Lauda-Königshofen, Germany) to 5 °C. The alcohol content was recorded using an Anton Paar Density Meter DMA35 (Anton Paar, Graz, Austria). The low wines were stored at 4 °C, prior to further analysis.

### 2.8. Sensory Analysis with Quantitative Descriptive Analysis

A quantitative descriptive analysis (QDA) was performed, as described in the literature [38,39,40]. The samples were assessed by The Scotch Whisky Research Institute (SWRI) expert sensory panel, consisting of 12 panellists over the age of 18, of mixed genders and ages. The training concentrated on the flavours related to whisky, centred around the SWRI Flavour Wheel, and being familiarised with a range of sensory techniques. The panel performance is assessed regularly by participation in the FlavorActiV Whisky Sensory Proficiency Scheme (https://www.flavoractiv.com/; accessed on 6 November 2021). The panel leader judged the performance of the panellists by their having acquired a suitable level of expertise, based on individual performance, in relation to the panel mean over several weeks.

The spirit samples were diluted to a uniform alcohol strength of 20% ABV using water and were then encoded with a three-digit code and analysed in a randomised order. The sensory attributes were preselected based on descriptors from the SWRI Flavour Wheel and previous experiences of new-make whisky spirits: *soapy*, *spicy*, *sour*, *sulphury*, *meaty*, *stale*, *feinty*, *stale*, *cereal*, *green*/*grassy*, *floral*, *fruity*, *solventy*, and *sweet*. The panellists scored the intensity of these attributes on a line scale from 0 to 3. The sensory assessment was only based on aroma and no tasting was carried out, which is the typical standard industry practise for laboratory spirits. The data collection was split into two sessions to reduce sensory fatigue. An amount of 20 mL of the samples was presented in 100 mL blue nosing glasses, covered with a cover glass, and prepared at least 30 min before nosing. The data were collected using Compusense software (West Guelph, Canada).

### 2.9. Congener Measurements by Gas Chromatography–Mass Spectrometry

A GC System 7890A (Agilent Technologies, Santa Clara, CA, USA), with a PAL RTC autosampler (PAL System, Zwingen, Switzerland), and an MS 5975C inert XL MSL with a Triple-Axis detector (Agilent Technologies, Santa Clara, CA, USA), with a DB WAX-UI column of 60 m, 0.32 mm, and 0.50 µm (Agilent, Santa Clara, CA, USA), was used to analyse the spirits. An amount of 2 mL of the spirit was filled into 10 mL headspace crimp top vials with 20 magnetic composite caps (Thermo Scientific, Waltham, MA, USA). An additional 0.5 mL was used to adjust the ABV to 20% with an ethanol and water mixture. Methyl heptanoate (50 µL, 20.5 ppm) (Sigma-Aldrich, St. Louis, MO, USA) was used as an internal standard. Spirits were prepared at least 24 h prior to analysis. Each fermentation was assessed in duplicate, resulting in six measurements per condition.

Samples were incubated for 5 min at 50 °C, and a DVB/Carbon WR/PDMS SMPE arrow fibre (Agilent, Santa Clara, NC, USA) was used to extract the volatiles for 10 min at 250 rpm and 50 °C. It was injected in a pulsed splitless mode, with a pressure of 21 psi for 3 min, followed by a purge flow of 50 mL/min to the column, with an injector temperature of 250 °C, and a desorption time of 15 min. The temperature of the column was set for 3 min at 35 °C, with a temperature increase rate of 10 °C/min to 240 °C, and this temperature was held for an additional 6 min, with a flow rate of 1.4 mL/min, resulting in a total run time of 29.5 min. The detector was set to 250 °C. A full scan was conducted, with a solvent delay of 1 min, and an m/z between 35 and 350.

A total of 230 components were identified with the MassHunter Workstation Software Quantitative Analysis Version B.07.01/Build 7.1.524.0 Unknown Analysis, 2008 (Agilent Technologies, Santa Clara, CA, USA), and the NIST/EPA/NIH Mass Spectral Library Version 2.2, built 10 January 2013 7 (National Institute of Standards and Technology, Gaithersburg, MD, USA). The peak areas were semiquantitatively analysed with MassHunter Workstation Software Quantitative Analysis Version B.07.01/Build 7.1.524.0 for GCMS (Agilent Technologies, Santa Clara, CA, USA) by comparing peak areas, but no standard or calibration lines were created. The flavour descriptors given on the Good Scents Company website http://www.thegoodscentscompany.com/ (accessed on 6 November 2021) were grouped according to the flavour attributes of the Scotch Whisky Flavour Wheel. Compounds without listed descriptors were assigned as “not described”.

### 2.10. Statistical Analysis

The statistical analysis was conducted with JMP 14.3.0 software (32-bit, SAS Institute Inc., Cary, NC, USA). The fermentation, flow cytometer, and QDA data were analysed with a two-way ANOVA, followed by a multiple comparison Tukey–Kramer HSD test. A *p*-value < 0.05 was taken as a statistically significant difference. The fermentation data (cell count, pH, FG (final gravity), ABV, growth phases), and the mean panel scores for all 14 sensory attributes, were further summarised by multiple factor analysis (MFA). To only show the most important components in order to separate the different treatment conditions, predictor screening was performed, with the selection of all components that had an influence larger than 3% on one of the conditions. The screening was performed five times, and all of the components that at least surpassed the threshold twice were used to create a heatmap by hierarchical clustering by Ward’s method on the standardized data.

## 3. Results

### 3.1. Effect of Pretreatments on Wort Quality

The studies showed that the pretreatment of wort prior to fermentation alters the wort composition and affects the fermentation performance in various ways. Parameters, such as the original gravity, FAN, and the concentration of higher alcohols, esters, and fatty acids could be decreased, while the protein, nitrogen, and carbonyl compounds increased [41]. While the wort used in this study was frozen prior to treatment, this process step has a limited effect on the composition. Both wild yeast and lactic acid bacteria have a high resistance to freezing, resulting in activities of around 90% after freezing [42,43,44,45,46,47,48] and, thus, the microbial stability is not impacted. The pretreatment of wort may be able to produce more reproducible wort for subsequent small-scale fermentation and reduce the impact of the active processes during fermentation not directly related to yeast. Therefore, we report various scenarios likely to be found when different treatments are given to wort prior to fermentation.

### 3.2. Effect of Filtration on Wort Quality

**Figure 1:**
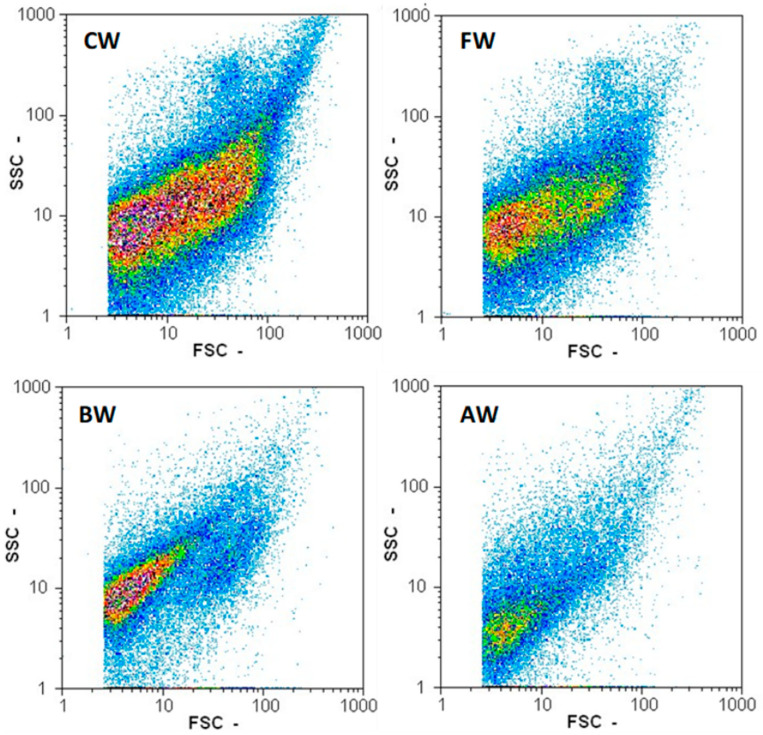
Flow cytometer particle count for the forward scatter (FSC) and the sideward scatter (SSC) for four different worts: CW, control wort; FW, filtered wort; BW, boiled wort; and AW, autoclaved wort.

**Figure 2:**
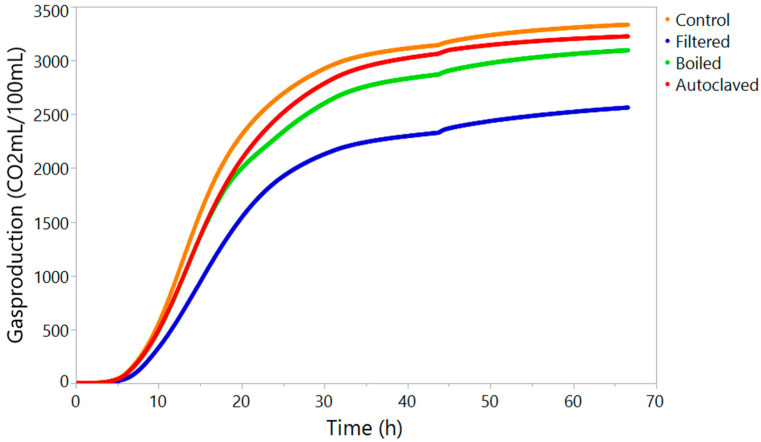
Ankom gas production curve over 68 h for four pretreated worts inoculated with the same yeast.

**Figure 3:**
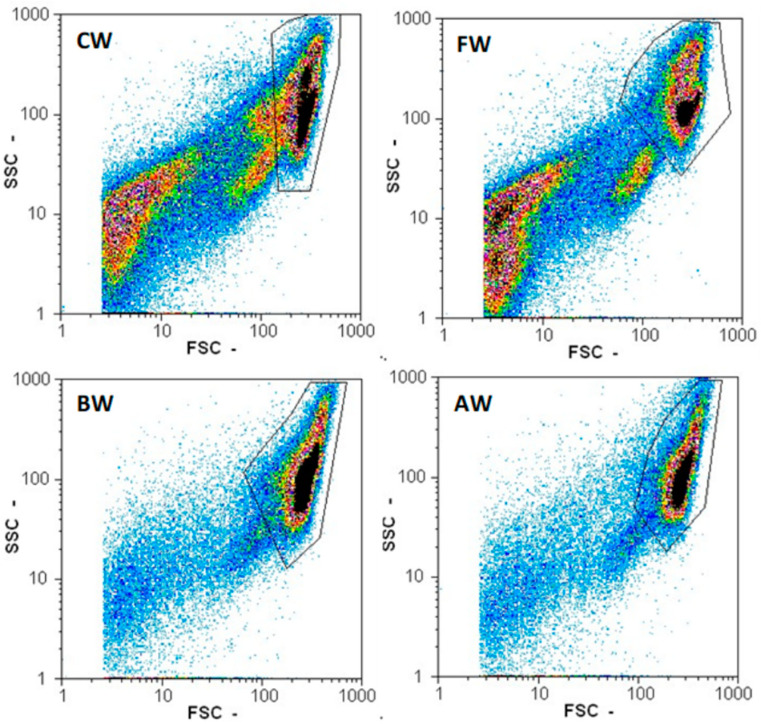
Flow cytometer particle counts for the forward scatter (FSC) and the sideward scatter (SSC) of four fermentations with different pretreated wort: CW, control wort; FW, filtered wort; BW, boiled wort; and AW, autoclaved wort. Black boxes display the area that was considered for the yeast cell count and viability. Darker colours indicate a higher count in the area of the graph.

Filtration is often used to eliminate unwanted components by size, such as unwanted organisms. Figure 1 shows that the particle count of the filtered wort was reduced to half of the control wort. It had an overall reduction of particle counts, with mostly residual small particles. Table 1 summarises the overall performance of the yeasts in the different pretreated wort. However, a closer look at the results presented in Table 1 shows that the control wort had the highest ABV, produced the highest CO_2_ during fermentation, and had the lowest yeast viability in the wash, most likely due to the production of high alcohol levels and the harsher environment. Filtering the wort prior to fermentation resulted in a negative influence on the fermentation performance (Table 1, Figure 2), with a reduced ABV and final CO_2_ concentration. This was also seen in the increase of the FG compared to the value obtained from the control wort, suggesting that not all of the carbohydrates were converted to ethanol. An FG of 1015.3° is on the high side for an acceptable range for fermentation, which represents a loss of around 19.4 LPA/t (litres of pure alcohol). In addition, the yeasts reached different growth phases later than the control wort, and the yeast cellular morphology was impacted by being less elongated compared to the control wort (Figure 3).

**Figure 4:**
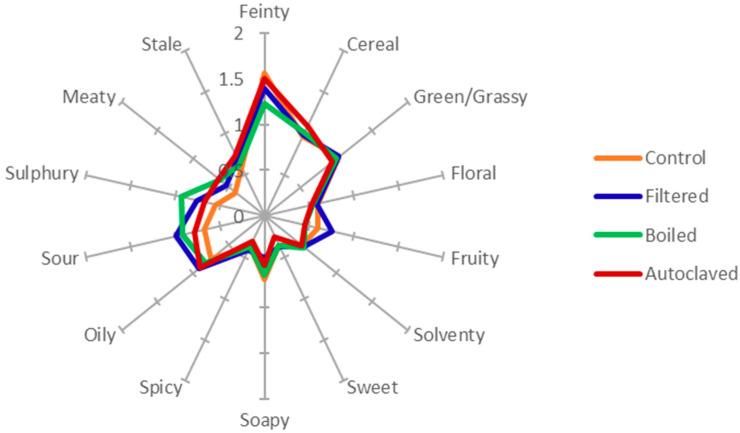
Spider diagram of the QDA mean panel scores for spirits of four pretreatment conditions for wort.

**Figure 5:**
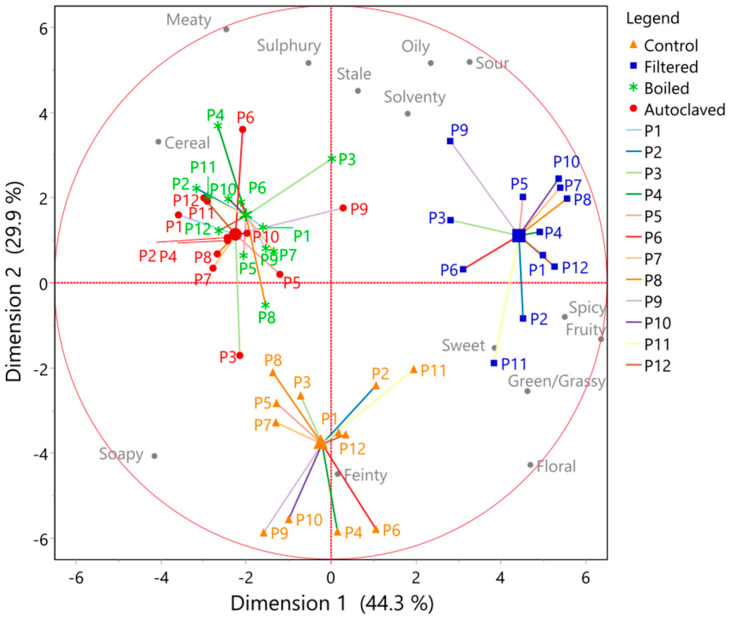
MFA plot of the QDA data, resulting from 12 panellists, for spirits of four pretreatment conditions for wort MFA analysis, for each panellist first.

**Figure 6:**
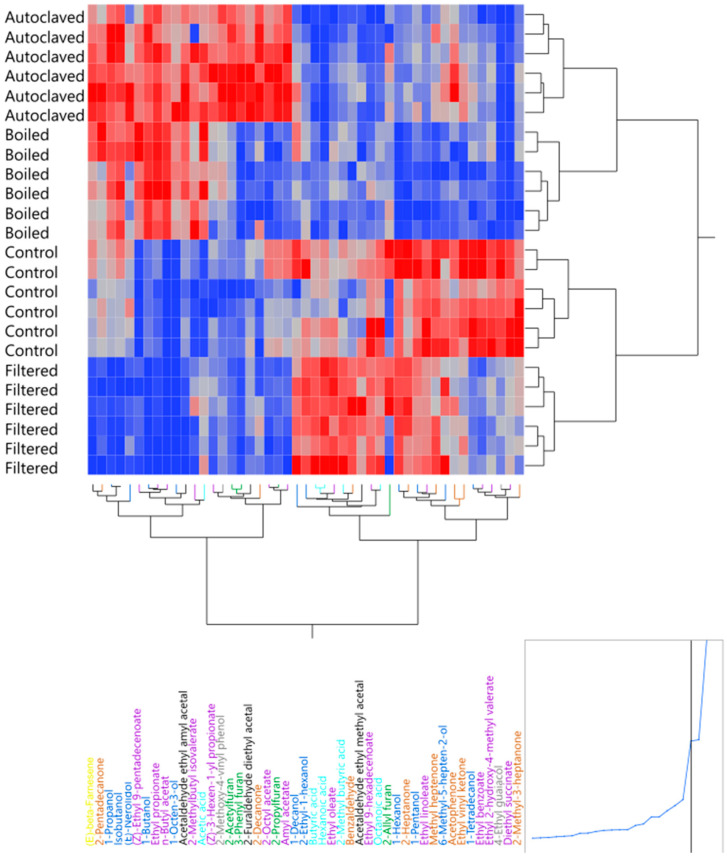
Heatmap of selected congener that drove the separation between the pretreatment techniques.

With regard to the flavour changes in the low-wine spirits, no significant or clear-cut differences were detected, as indicated in Figure 4. The observations with regard to the response of the individual panellists to the MFA is shown in Figure 5. Here, the consensus plot shows that the filtered wort was often described in higher strengths for a variety of lighter flavour attributes, such as *floral*, *green*/*grassy*, *sweet*, *fruity*, *spicy*, *solventy*, *oily*, and *sour* (Figure 6) (see also additional comments in Table 2). This shows that all of the panellists separated the spirits from each other; however, because of the nature of low wines, the panellists could not agree which parameter separated the presented spirits.

**Figure 7:**
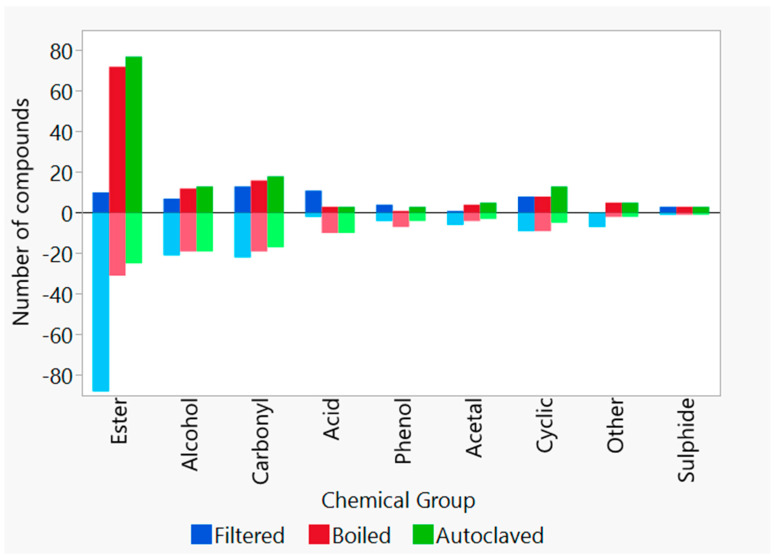
Number of compounds that showed an increased or decreased peak area, in comparison to the control wort, separated by the main nine chemical groups.

The compositional analysis of congeners by GC-MS (Figure 6) shows that, overall, the concentration of the most detected esters, higher alcohols, carbonyl compounds, acetals, and others were reduced compared to the control wort. However, the levels of acids and sulphides increased compared to the control wort. By selecting the congeners that predominantly impact the separation between groups (Figure 6 and Figure 7), the filtered wort had lower peak areas for esters and higher alcohols, while the peak areas for the acids and curtained congeners of each group were increased. The few esters that increased belonged to the group of fatty acid ethyl esters. The decrease in esters and higher alcohols was an unexpected result because the presence of lactic acid bacteria in whisky is often connected to higher acid and ester concentrations [2,3,4,5,6,7,18,23,49,50]. Nevertheless, less turbid worts are linked to a reduction in fatty acids [51], which are needed for ester production and explains their low concentration.

While higher acid concentrations were detected in the filtered wort, and the pH was significantly lower than the control wort, the filtered wort had the lowest peak areas for most esters. Moreover, on the basis of the lowest levels of esters and higher alcohols, a heavier character was expected than the lighter character suggested by the MFA analysis. Nevertheless, the GC-MS analysis did only document peak areas, and not congener concentration, which makes it difficult to correlate the flavour directly to the GC-MS data. While the peak areas for the filtered wort are lower for the esters, they are already odour-active in low concentrations, resulting in fruity floral effects on the spirit. The nutrients in wort that contribute to wort solids, such as proteins or peptides, lipids, organic acids, polyphenols, and calcium oxalate and β-glucan, which result in flavour precursors [52,53,54,55], were reduced by the filtration step, resulting in an overall reduction of light congeners, such as esters and higher alcohols.

The lipid content of the wort impacted the congener profile. High concentrations of unsaturated fatty acids, such as oleic, linoleic, and linolenic acid, can reduce ester synthesis [56,57]. The frozen and filtered wort esters of these fatty acids (ethyl oleate, ethyl linoleate), and fatty acids (lauric acid, octanoic acid, butyric acid, isobutyric acid, propanoic acid) increased. Other potential factors influencing flavour changes are different yeast stress responses, the presence of other microorganisms, and lowered fermentation pH [58]. The change in yeast cellular morphology (Figure 4) additionally points to adverse changes in the fermentative metabolism.

### 3.3. Effect of Boiling and Autoclaving on Wort Quality

**Figure 8:**
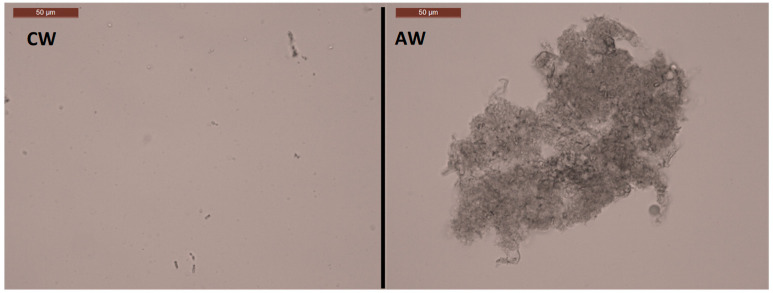
Example microscope pictures of non- and heat-treated wort: Left, control wort (CW); Right, autoclaved wort (AW).

Wort boiling is a typical approach in brewing used to sterilize the wort, coagulate proteins, and inactivate enzymes. This influences the congener concentrations, acidifies the wort, and reduces the water [59]. In contrast, in the distilling industry, wort boiling is not part of the process, as distillation is carried out after fermentation to produce the spirit. Autoclaving is, in comparison, a harsher heat-treatment method. Both treatments show similar traits, and the influence of both methods on wort was investigated. The boiling of wort reduced the total particle count in the wort to lower than one-third, compared to the control wort consisting mostly of particles around the size of 10SSC/3FSC, and a second group around the size of 10SSC/30FSC (Figure 1). Autoclaving the wort had the largest impact on the primary particle count by reducing it to one-fourth, compared to the control wort consisting mostly of small particles around 3SSC/3FSC (Figure 1). Microscopic assessment of the samples prior to assessment by flow cytometry showed that larger agglomerations of particles were present in the boiled and autoclaved wort of the size of around 150 µm (Figure 8). During wort boiling, the proteins from the malted barley coagulate with polyphenols and form insoluble coagulates, resulting in reduced protein, amino acid, and nitrogen contents [59,60].

As for the filtered wort, boiling and autoclaving had a negative impact on the fermentation performance (Table 1). The ABV and final CO_2_ concentration decreased, with a loss of at least 6.2 LPA/t. There was no impact on the yeast reaching the different growth phases, only that the cumulative CO_2_ concentrations at these points were lower, showing that the fermentation is not as efficient (Figure 2, Table 1). For Scotch whisky fermentation, the activity of α-amylase, β-amylase, limit dextrinase, and α-glucosidase during fermentation is important for the breakdown of the residual starch and a gain of high alcohol yields [10]. These enzymes would be inactivated by heat pretreatments.

The total particle count, after fermentation, was reduced early, ten-fold, with a clear reduction in the small particles where indigenous microorganisms are measured (Table 1, Figure 3). Additionally, more yeasts remained viable at the end of the fermentation (Table 1). By boiling/autoclaving wort, enzymes were not only deactivated, but microorganisms were also killed, reducing the competition and stress on the yeast during fermentation, in addition to preventing the pH dropping as low as in the control wort [58].

With regard to flavour changes in the low-wine distillates (Figure 4), no significant differences could be measured. However, the consensus plot of the MFA showed that the panellists, excluding Panellist Eight, scaled the samples differently from the control (Figure 5). The samples were mostly described by more *feinty* characteristics, such as *meaty*, *cereal*, or *sulphury*. Unexpectedly, these samples had higher peak areas for most esters, as well as higher alcohols, carbonyl, sulphides, acids, and arenes (Figure 6 and Figure 7). During wort boiling, two main reactions influence the flavour and when congener composition takes place: the Maillard reaction and Strecker degradation. One of these compounds is 2-acetylfuran [59], which is one of the compounds that were a major factor separating the four different wort treatment conditions. In this case, the compounds produced during the wort boiling may mask the flavour of higher alcohols and esters, resulting in an overall heavier spirit character.

## 4. Conclusions

This study investigated the impact of wort pretreatments on fermentation performance and changes in the flavour and congener profile of Scotch whisky low wines. No significant differences could be detected by nosing low wines, while the chemical compositions showed clear changes in the increased levels of esters and higher alcohols in boiled and autoclaved wort, while the filtered wort had an overall decrease in congeners. The treatment resulted in a lower alcohol yield. The pretreatment of wort prior to fermentation will require additional process operations. Sterilizing wort would eliminate the need for keeping it frozen, but changes in congeners inevitably occur, as seen from the sensory analysis from this study. This results in relating the fermentation performance to whisky production more difficult. This study has revealed that experimental fermentation studies should use either fresh or frozen wort to present meaningful data for the distilling industry. A comparison of different options is presented in Table 2.

## Figures and Tables

**Table 1:** Average and standard deviations of fermentation parameters after fermentation for three pretreatment conditions for wort compared to a control wort. Letters (a, b, c) indicate significant differences (*p* < 0.05).

	Control	Filtered	Boiled	Autoclaved
FG (°)	1000.7 ± 0.8 ^c^	1015.3 ± 0.8 ^a^	1005.3 ± 0.6 ^b^	1005.4 ± 0.2 ^b^
pH	3.90 ± 0.00 ^b^	3.75 ± 0.04 ^c^	4.54 ± 0.00 ^a^	4.56 ± 0.01 ^a^
ABV (%)	9.2 ± 0.1 ^a^	7.2 ± 0 ^c^	8.4 ± 0 ^b^	8.5 ± 0 ^b^
End of lag phase (h)	7.6 ± 0.1 ^b^	8.03 ± 0.15 ^a^	7.63 ± 0.05 ^b^	7.63 ± 0.15 ^b^
Vmax (h)	13 ± 0.08 ^b^	14.94 ± 1.1 ^a^	13.44 ± 0.17 ^b^	13.64 ± 0.19 ^a,b^
Start of stationary phase (h)	19.69 ± 0.04 ^c^	22.33 ± 0.25 ^a^	19.11 ± 0.29 ^c^	21.33 ± 0.36 ^b^
Cumulative CO_2_ production (mL/100 mL)	3332.82 ± 56.52 ^a^	2563.09 ± 27.65 ^c^	3095.57 ± 36.94 ^b^	3225.02 ± 153.01 ^a,b^
Total count (Count/mL)	1.00 × 10^8^ ± 2.06 × 10^6 a^	8.72 × 10^7^ ± 9.48 × 10^6 a^	2.62 × 10^7^ ± 1.22 × 10^7 c^	4.81 × 10^7^ ± 4.45 × 10^6 b^
Yeast count (Count/mL)	4.33 × 10^7^ ± 2.66 × 10^5 a^	2.55 × 10^7^ ± 1.71 × 10^6 b,c^	1.93 × 10^7^ ± 1.02 × 10^7 c^	3.88 × 10^7^ ± 2.40 × 10^6 a,b^
Alive yeast (%)	51.6 ± 4.4 ^b^	55.4 ± 0.6 ^a,b^	63 ± 3.4.0 ^a^	60.2 ± 2.0 ^a^
Dead yeast (%)	40.6 ± 4.2 ^a^	34.4 ± 0.5 ^a^	24.8 ± 3.2 ^b^	26.9 ± 1.0 ^b^
Inactive yeast (%)	3.8 ± 0.1 ^b^	3.7 ± 0.4 ^b^	8.7 ± 0.3 ^a^	8.5 ± 0.1 ^a^

**Table 2:** Summary of the advantages and disadvantage of wort storage and treatment methods.

Wort Stage	Advantages	Disadvantages
Fresh Industry	as close to industry as possible no influence on microorganisms or enzymes	cannot be stored not stable difficult to obtain
Fresh Lab-Scale	easy to obtain right batch size producible on the same day	small batches microflora other than industry wort
Frozen	possible long-time storage provides the possibility of using the same wort for several fermentations	reduction of microorganism needs freezer space
Filtered		reduction of congener precursor difficult to reduce microorganisms by filtering loss of alcohol
Boiled	easy sterile	agglomeration of enzymes sterile loss of alcohol
Autoclaved	only applicable in the lab sterile	agglomeration of enzymes sterile loss of alcohol

## Data Availability

The authors confirm that the data supporting the findings of this study are available within the article.

## References

[1] (2009). Scottish Government, The Scotch Whisky Regulations. http://www.legislation.gov.uk/uksi/2009/2890/pdfs/uksi_20092890_en.pdf.

[2] Guild I.B., Street H., Simmonds P.G.W., Lloyd W.J.W. (1985). The Sections. J. Inst. Brew..

[3] Van Beek S., Priest F.G. (2002). Evolution of the Lactic Acid Bacterial Community during Malt Whisky Fermentation: A Polyphasic Study. Appl. Environ. Microbiol..

[4] Reid S.J., Speers R.A., Willoughby N., Lumsden W.B., Maskell D.L. (2020). Pre-Fermentation of Malt Whisky Wort Using *Lactobacillus plantarum* and Its Influence on New-Make Spirit Character. Food Chem..

[5] Lowe D.P., Arendt E.K. (2004). The Use and Effects of Lactic Acid Bacteria in Malting and Brewing with Their Relationships to Antifungal Activity, Mycotoxins and Gushing: A Review. J. Inst. Brew..

[6] Vriesekoop F., Krahl M., Hucker B., Menz G. (2012). 125th Anniversary Review: Bacteria in Brewing: The Good, the Bad and the Ugly. J. Inst. Brew..

[7] Barbour E.A., Priest F.G. (1988). Some Effects of *Lactobacillus* Contamination in Scotch Whisky Fermentations. J. Inst. Brew..

[8] Simpson K.L., Pettersson B., Priest F.G. (2001). Characterization of *Lactobacilli* from Scotch Malt Whisky Distilleries and Description of *Lactobacillus ferintoshensis* sp. nov., a New Species Isolated from Malt Whisky Fermentations. Microbiology.

[9] Bringhurst T.A., Brosnan J., Russell I., Stewart G.G. (2014). Scotch Whisky: Raw Material Selection and Processing. Whisky Technology, Production and Marketing.

[10] Bathgate G.N. (2016). A Review of Malting and Malt Processing for Whisky Distillation. J. Inst. Brew..

[11] Vriesekoop F., Rathband A., MacKinlay J., Bryce J.H. (2010). The Evolution of Dextrins during the Mashing and Fermentation of All-Malt Whisky Production. J. Inst. Brew..

[12] Walker J.W., Bringhurst T.A., Broadhead A.L., Brosnan J.M., Pearson S.Y. (2001). The Survival of Limit Dextrinase during Fermentation in the Production of Scotch Whisky. J. Inst. Brew..

[13] Sim G.B., Berry D.R. (1996). Malted Barley Enzyme Activity under Optimum and Process Conditions from the Scotch Malt Whisky Industry. Enzyme Microb. Technol..

[14] Evans E., Van Wegen B., Ma Y., Eglinton J. (2004). The Impact of the Thermostability of α-Amylase, β-Amylase, and Limit Dextrinase on Potential Wort Fermentability. J. Am. Soc. Brew. Chem..

[15] Watson D.C. (1981). The Development of Specialised Yeast Strains for Use in Scotch Malt Whisky Fermentations. Advances in Biotechnology.

[16] Stewart G.G., Hill A.E., Russell I. (2013). 125th Anniversary Review: Developments in Brewing and Distilling Yeast Strains. J. Inst. Brew..

[17] Walker G., Brosnan J., Bringhurst T., Jack F., Goodall I., Fotheringham R., Murray D.R.A., Walker G.M. (2011). Selecting New Distilling Yeasts for Improved Fermentation and for Sustainability. Distilled Spirits—Future Challenges, New Solutions.

[18] Walker G., Hill A. (2016). Saccharomyces Cerevisiae in the Production of Whisk(e)Y. Beverages.

[19] Kyraleou M., Herb D., Reilly G.O., Conway N., Bryan T., Kilcawley K.N. (2021). The Impact of Terroir on the Flavour of Single Malt Whisk(e)y New Make Spirit. Foods.

[20] Master of Malt Glen Elgin 18 Year Old 1998 (Special Release 2017). https://www.masterofmalt.com/whiskies/glen-elgin/glen-elgin-18-year-old-1998-special-release-2017-whisky/.

[21] Broom D. (2019). Is Yeast Whisky’s New Frontier of Flavour?. https://scotchwhisky.com/magazine/features/22834/is-yeast-whisky-s-new-frontier-of-flavour/.

[22] Ramsay C.M., Berry D.R. (1983). Development of a Small Scale Mashing and Fermentation System for Studies on Malt Whisky Production. Eur. J. Appl. Microbiol. Biotechnol..

[23] Dolan T.C.S. (1976). Some Aspects of the Impact of Brewing Science on Scotch Malt Whisky Production. J. Inst. Brew..

[24] European Brewing Convention (EBC) (1997). 4.6.1—Hot Water Extract of Malt: Constant Temperature Mash Analytica EBC. BrewUp.

[25] Yonezawa T., Stewart G.G., Bryce J.H., Stewart G.G. (2004). Monitoring and Controlling of Whisky Fermentation. Distilled Spirits—Tradition and Innovation.

[26] Ramsay C.M., Berry D.R. (1984). The Effect of Inoculum Level on the Formation of Higher Alcohols, Fatty Acids and Esters in the Malt Whisky Fermentation. Food Microbiol..

[27] Wanikawa A., Hosoi K., Kato T. (2000). Conversion of Unsaturated Fatty Acids to Precursors of γ-Lactones by Lactic Acid Bacteria during the Production of Malt Whisky. J. Am. Soc. Brew. Chem..

[28] Ensor M., Bryce J.H., Hill A., Goodall I., Fotheringham R., Murray D.R.A., Walker G.M. (2015). An Investigation into the Use of Different Yeast Strains and Lactobacillus on New Make Spirit. Distilled Spirits—Future Challenges, New Solutions.

[29] Neto H.B.d.A., Pearson S.Y., Walker J.W., Walker G.M., Brosnan J., Bryce J.H., Piggott J.R., Stewart G.G. (2008). Application of Nocel Yeast Strains to the Scotch Whisky Fermentation Process. Distilled Spirits, Production, Technology and Innovation.

[30] Berbert de Amorim Neto H., Yohannan B.K., Bringhurst T.A., Brosnan J.M., Pearson S.Y., Walker J.W., Walker G.M. (2009). Evaluation of a Brazilian Fuel Alcohol Yeast Strain for Scotch Whisky Fermentations. J. Inst. Brew..

[31] Storr A., Walker J.W., Jack F., Dabrowska D., Davies S., Garden M., Maskell D., Murray D. (2018). Assessment of Pinnacle MG+ Yeast for the Scotch Whisky Industry. Distilled Spirits: Local Roots; Global Reach: Delivering Distilling Expertise to the World.

[32] Lei H., Zhao H., Yu Z., Zhao M. (2012). Effects of Wort Gravity and Nitrogen Level on Fermentation Performance of Brewer’s Yeast and the Formation of Flavor Volatiles. Appl. Biochem. Biotechnol..

[33] Larson J.W. (2014). Designing for Cleanliness in the Distillery. Whisky.

[34] Makanjuola D.B., Tymon A., Springham D.G. (1992). Some Effects of Lactic Acid Bacteria on Laboratory-Scale Yeast Fermentations. Enzyme Microb. Technol..

[35] Wilson N. (2014). Contamination: Bacteria and Wild Yeasts in a Whisky Fermentation. Whisky.

[36] HM Revenue & Customse Notice 226: Beer Duty. https://www.gov.uk/government/publications/excise-notice-226-beer-duty/excise-notice-226-beer-duty--2.

[37] Black K., Daute M., Tziboula-Clarke A., White P.J., Iannetta P.P.M., Walker G. (2021). Utilization of Low Nitrogen Barley for Production of Distilling Quality Malt. J. Am. Soc. Brew. Chem..

[38] Lawless H.T., Heymann H., Heldman D.R. (2010). Sensory Evaluation of Food.

[39] Stone H., Sidel J., Oliver S., Woolsey A., Singleton R.C. (1974). Sensory Evaluation by Quantitative Descriptive Analysis. Food Technol..

[40] Daute M., Jack F., Baxter I., Harrison B., Grigor J., Walker G. (2021). Comparison of Three Approaches to Assess the Flavour Characteristics of Scotch Whisky Spirit. Appl. Sci..

[41] Štěrba K., Čejka P., Olšovská J. (2020). Influence of Beer and Wort Sample Freezing on the Result of Analysis. Kvas. Prum..

[42] Mazur P., Schmidt J.J. (1968). Interactions of Cooling Velocity, Temperature, and Warming Velocity on the Survival of Frozen and Thawed Yeast. Cryobiology.

[43] Weiser R.S., Hargiss C.O. (1946). Studies on the Death of Bacteria at Low Temperatures. J. Bacteriol..

[44] Kandil S., El Soda M. (2015). Influence of Freezing and Freeze Drying on Intracellular Enzymatic Activity and Autolytic Properties of Some Lactic Acid Bacterial Strains. Adv. Microbiol..

[45] Bravo-Ferrada B.M., Brizuela N., Gerbino E., Gómez-Zavaglia A., Semorile L., Tymczyszyn E.E. (2015). Effect of Protective Agents and Previous Acclimation on Ethanol Resistance of Frozen and Freeze-Dried *Lactobacillus plantarum* Strains. Cryobiology.

[46] De Giulio B., Orlando P., Barba G., Coppola R., De Rosa M., Sada A., De Prisco P.P., Nazzaro F. (2005). Use of Alginate and Cryo-Protective Sugars to Improve the Viability of Lactic Acid Bacteria after Freezing and Freeze-Drying. World J. Microbiol. Biotechnol..

[47] Cabrera E., Welch L.C., Robinson M.R., Sturgeon C.M., Crow M.M., Segarra V.A. (2020). Cryopreservation and the Freeze–Thaw Stress Response in Yeast. Genes.

[48] Dumont F., Marechal P.A., Gervais P. (2004). Cell Size and Water Permeability as Determining Factors for Cell Viability after Freezing at Different Cooling Rates. Appl. Environ. Microbiol..

[49] Wanikawa A., Hosoi K., Takise I., Kato T. (2000). Detection of γ-Lactones in Malt Whisky. J. Inst. Brew..

[50] Wanikawa A. (2020). Flavors in Malt Whisky: A Review. J. Am. Soc. Brew. Chem..

[51] Kühbeck F., Back W., Krottenthaler M. (2006). Influence of Lauter Turbidity on Wort Composition, Fermentation Performance and Beer Quality -in Large-Scale Trials. J. Inst. Brew..

[52] MacWilliam I.C. (1968). Wort Compostion—A Review. J. Inst. Brew..

[53] Freeman G.J., McKechnie M.T., Lea A.G.H., Piggott J.R. (2003). Filtration and Stabilization of Beers. Fermented Beverage Production.

[54] Bamforth C.W. (2008). Wort Composition and Beer Quality. Brewing Yeast Fermentation Performance.

[55] Schneider J., Krottenthaler M., Back W., Weisser H. (2005). Study on the Membrane Filtration of Mash with Particular Respect to the Quality of Wort and Beer. J. Inst. Brew..

[56] Peddie H.A.B. (1990). Ester Formation in Brewery Fermentations. J. Inst. Brew..

[57] Saerens S.M.G., Delvaux F.R., Verstrepen K.J., Van Dijck P., Thevelein J.M., Delvaux F.R. (2008). Parameters Affecting Ethyl Ester Production by *Saccharomyces cerevisiae* during Fermentation. Appl. Environ. Microbiol..

[58] Deak T., Rosa C.A., Peter G. (2006). Environmental Factors Influencing Yeasts. Biodiversity and Ecophysiology of Yeasts.

[59] Willaert R.G., Baron G.V. (2001). Wort Boiling Today-Boiling Systems with Low Thermal Stress in Combination with Volatile Stripping. Cerevisia.

[60] Bei J., Lin L., Guo-Qin L., Bing L., Yu-Kui Z., Liao-Ning L. (2009). Structural Changes of Malt Proteins during Boiling. Molecules.

